# Enhancing Efficiency in Post-Anesthesia Care Unit Discharges: A Non-Clinical Audit Perspective

**DOI:** 10.12669/pjms.41.11.13137

**Published:** 2025-11

**Authors:** Faraz Mansoor, Robina Bangash, Salma Jan, Zain ul Abidin

**Affiliations:** 1 Faraz Mansoor, FCPS (Anesthesia), FCPS (Critical Care Medicine). Consultant Anesthetist, Department of Anesthesia, Shaukat Khanum Memorial, Cancer Hospital and Research Center, Peshawar, Pakistan; 2 Robina Bangash, FCPS. Consultant Anesthetist, Department of Anesthesia, Shaukat Khanum Memorial, Cancer Hospital and Research Center, Peshawar, Pakistan; 3 Salma Jan, FCPS. Consultant Anesthetist, Department of Anesthesia, Shaukat Khanum Memorial, Cancer Hospital and Research Center, Peshawar, Pakistan; 4 Zain ul Abidin, FCPS. Consultant Anesthetist, Department of Anesthesia, Shaukat Khanum Memorial, Cancer Hospital and Research Center, Peshawar, Pakistan

**Keywords:** Post-Anesthesia Care Unit, Length of Stay, Audit, Key Performance Indicator

## Abstract

**Background and Objective:**

Prolonged stays in the Post-Anesthesia Care Unit (PACU) can negatively impact patient safety, hospital length of stay, and surgical workflow. While clinical reasons for delayed discharge are often studied, non-clinical factors such as bed unavailability and staff busyness are less frequently addressed. Our objective was to identify non-clinical factors contributing to delayed PACU discharge and implement quality improvement strategies to reduce PACU length of stay.

**Methodology:**

A new departmental Key Performance Indicator (KPI) was introduced in July 2023 to monitor PACU length of stay, with “delayed discharge” defined as exceeding one-hour post-fitness for discharge. An audit was conducted using retrospective data from 69 patients in September 2023. Two Plan-Do-Study-Act (PDSA) cycles were conducted. Interventions included regular educational sessions, expanding the audit team, initiating monthly departmental quality improvement meetings, creating a new data collection form to assess staff busyness, and adding two beds to the High Dependency Unit (HDU).

**Results:**

Baseline data showed that only 62% of patients were discharged within the target timeframe. After the first PDSA cycle, compliance improved to 74%. Following the second PDSA cycle, supported by multidisciplinary collaboration and infrastructure improvements, the department achieved its target (≥95%) by February 2024. Contributing non-clinical factors leading to delayed discharges included staff busyness (e.g., shift changes, porter delays) and lack of available beds.

**Conclusion:**

Addressing non-clinical factors such as staffing logistics and bed availability significantly improved PACU discharge efficiency. Structured quality improvement initiatives and interdepartmental collaboration were key to achieving sustained improvements in patient flow and departmental performance.

## INTRODUCTION

One of the primary objectives of the Post-Anesthesia care unit (PACU) is to deliver continuous, round-the-clock care to patients with the goal of preventing, identifying, and promptly treating the complications. The specific configuration and design of the PACU typically align with the patient demographic served by a given healthcare institution. Notably, the PACU may, on occasion, accommodate non-surgical patients, particularly in instances where there is shortage of beds in the intensive care unit (ICU). For example, in the recent past a discernible surge has been observed in the admission of patients afflicted with COVID-19 necessitating mechanical ventilation within the confines of the post-anesthesia care unit. Generally, the recommended ratio of beds in the Stage 1 Recovery is 2:1. In other words, two beds are required for one operating room. This capacity is crucial for efficient running of the operating room (OR). Some patients are directly transferred to the ICU after surgery.

Regrettably, consensus remains elusive within the healthcare community regarding the optimal duration of post-anesthesia care unit (PACU) stay. For the purpose of this audit, a protracted PACU discharge interval was operationally defined as exceeding one hour, indicative of the patient’s readiness for release.^1^ It is noteworthy to underscore that pertinent investigations have addressed both clinical and non-clinical determinants contributing to extended PACU stays.^2^

The determinants contributing to an extended PACU stay can be categorized into two distinct classes: clinical and non-clinical. Clinical factors encompassing nausea, vomiting, cardiovascular and respiratory complications, and challenges in pain management are identified as potential contributors to a prolonged PACU stay. Conversely, non-clinical factors commonly associated with delayed transfer from PACU involve bed unavailability and staff busyness. The duration of PACU stay is regarded as a clinical indicator.^3^ The main objective of this audit was to identify the non-clinical factors that can lead to a delayed discharge from the PACU.

### Rationale of the audit:

Undoubtedly, the protraction of patient discharges exerts significant repercussions on the healthcare system. Such delays can lead to prolonged hospital length of stay, jeopardize patient safety, escalate financial expenditures, and more importantly result in the cancellation of both elective and emergency surgical procedures. Nonetheless, diligent identification and systematic addressing of these challenges hold the potential to enhance the overall efficiency of the healthcare system.^4^

### Baseline data:

In July 2023, the Department of Quality and Patient Safety within the hospital initiated discussions with the anesthesia team regarding the implementation of a new key performance indicator (KPI). This proposition was made in light of the fact that the existing KPI, focusing on nausea and vomiting, consistently achieved mean/median values of 97%, thus meeting the established criteria. Following deliberations with the anesthesia team, it was collectively determined that monitoring the Post-Anesthesia Care Unit (PACU) length of stay would be a more pertinent metric.

This decision was motivated by the observation that certain patients were experiencing prolonged stays in the PACU, with a subset even surpassing one day. Such protracted stays were attributed to the unavailability of beds. Consequently, a new departmental indicator was officially instituted on the 28^th^ of July 2023 to gauge and address the PACU length of stay. After introducing the new KPI, baseline data was collected.

Subsequent to the implementation of new KPI focusing on PACU length of stay, baseline data were collected in September 2023 through a retrospective examination of medical records belonging to 69 patients. The analysis revealed that 26 of these patients experienced an extended duration of stay in the facility. Notably, nursing notes indicated that staff members were occupied; however, the precise cause of staff busyness remained indistinct.

### Strategy:

### PDSA Cycle one:

The inaugural Plan-Do-Study-Act (PDSA) cycle lasted from October to November, 2023. Firstly, a departmental meeting was convened. During this meeting, the baseline findings were presented, and healthcare staff were apprised of the significance of the new KPI. In addition to these weekly educational sessions were arranged for the anesthesia staff to create awareness about the quality improvement project and its importance. In addition to this, PACU staff was also asked to document the causes of the delayed discharges in their patient’s notes. Outcomes were assessed in the month of November. The data indicated a commendable enhancement in the compliance, rising from 62% to 74%. To elucidate, approximately two-thirds of cases demonstrated adherence to the stipulated PACU length of stay, specifically within a one-hour timeframe. This noteworthy improvement signified a substantial advancement in the department’s efficiency. However, it is noteworthy to mention that the established target set was 95% and the department was still falling short of this benchmark. In light of the persistent gap from the target, it was decided to continue the educational sessions and systematically review staff feedback regarding common causes of discharge delays. These insights would guide the design of targeted interventions in the subsequent improvement cycle.

### PDSA Cycle two:

In December 2023, a departmental meeting was organized during which the results of the initial PDSA cycle were shared. Additionally, the root causes contributing to delayed transfers from the PACU were communicated to the department for comprehensive understanding and collaboration in addressing the identified issues. In response to the imperative of enhancing the efficacy of our audit endeavors, a strategic decision was made to expand the audit team which involved the inclusion of a senior nurse specializing in Post-Anesthesia Care Unit (PACU), a clinical nurse manager and two senior nurses from the inpatient department, specifically designated as Registered Nurses at Level 3 (RN3). One of the main objectives of this decision was to harness a diverse array of clinical perspectives and expertise.

In addition, in order to institutionalize a structured approach towards continuous quality improvement, it was decided to conduct regular meetings on a monthly basis. The primary objective of these meetings was to document the audit findings and subsequently formulate targeted strategies aimed at optimizing the patient flow within our health facility. Moreover, a novel data collection form was introduced to precisely ascertain the factors contributing to staff busyness. This proactive measure was implemented to provide a more detailed analysis of the causes associated with staff workload, thereby facilitating informed decision making and targeted interventions.

**Table-I:** Summary of interventions and outcomes across two PDSA cycles to improve PACU discharge compliance.

Cycle	Plan	Do	Study	Act
PDSA Cycle 1 (Oct–Nov 2023)	Dept. meeting, shared baseline findings, set KPI target (95%)	Weekly education (anesthesia staff); PACU staff started documenting reasons of delays	Compliance improved from 62% to 74% (Nov 2023)	Continue education; collect staff feedback; plan targeted interventions
PDSA Cycle 2 (Dec 2023–Feb 2024)	Meeting on Cycle 1 results, discussed root causes	Expanded audit team (PACU nurse, RN3s); monthly meetings; new data form; two HDU beds added	Ongoing monitoring; progressive improvement	Target achieved (95%); sustained improvement

**Fig.1:**
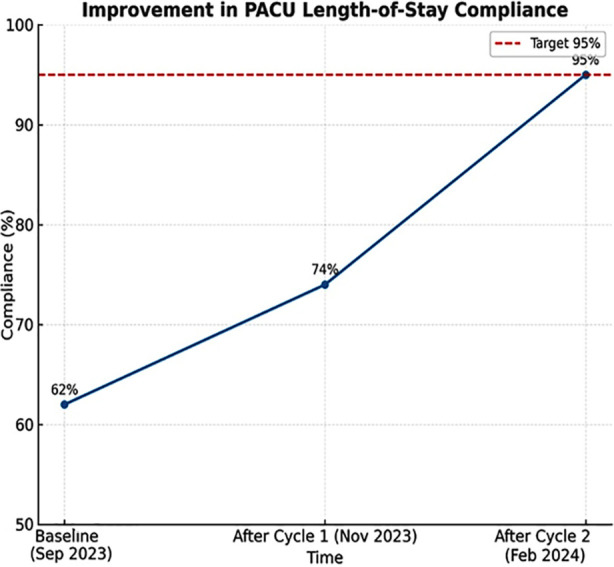
Compliance with PACU length-of-stay target before and after two PDSA cycles, showing progressive improvement from baseline (62%) to target achievement (95%).

During the same corresponding time frame, the hospital management established two additional beds in the High Dependency Unit to accommodate the post-operative surgical patients. This strategy continued during the months of January and February of the year 2024 and the results collected by the end of February indicated that the department had effectively achieved its targets. Fig.1 displays a line graph, and Table summarizes the main points of different components of the two Plan-Do-Study-Act (PDSA) cycles. Fig.1.

Top of Form

## DISCUSSION

Patients are kept in the post-anesthesia care unit after surgery for monitoring, prevention and management of complications, until they are ready to be discharged. This stay can be prolonged due to both clinical and non-clinical factors. Quite often, predominant emphasis is placed on the former, while the non-clinical factors are regrettably marginalized.

At the start of this audit, just 62% of the patients were meeting the target. However, this proportion was much higher than reported in a prospective observational study where 76% of the patients had a delayed discharge due to non-clinical factors.^5^ It was observed that a significant portion of the personnel involved in patient care within the Post-Anesthesia Care Unit and wards lacked awareness regarding the expeditious transfer of patients from the PACU. In order to enhance the discharge planning, all members of the multidisciplinary team should be made aware of the target discharge time. There is no doubt that the lack of communication between the care providers can jeopardize the patient care.^6^

In order to measure and monitor the operation room performance and efficiency, departments should develop a dashboard which could track a set of performance indicators.^7^ One of the paramount considerations in clinical practice is the identification of pertinent issues, followed by the establishment of benchmarks or Key Performance Indicators (KPIs), which subsequently facilitate systematic measurement. These quality indicators serve as invaluable tools for anesthesiologists, enabling them to engage in rigorous self-assessment process.^8^ Prior to their implementation, these benchmarks undergo scrutiny and approval by the hospital’s quality department. Initially, a benchmark of one hour for discharge from the PACU upon the patient’s deemed fitness for discharge was established for inpatients, while day cases were allotted 45 minutes for the same purpose. Subsequently, following deliberation with the hospital quality department, this policy was amended to unify the discharge timeframe to one hour for both inpatients and day cases.^1^

In this audit, it was observed that staff busyness and unavailability of the beds in the wards were the most common reasons for a delayed discharge of patients from the PACU. These findings align with an audit by Cobbe KA, which reported that bed unavailability was the second most common cause of delayed discharge.^5^ In this audit conducted at Shaukat Khanum Memorial Cancer Hospital, Peshawar, where new operating room services were established, the unavailability of ward beds was attributed to the saturation of hospital beds, whereby patients within the ward areas awaited discharge before accommodating new patients from the operating theater. This situation latter improved with the help of monthly team meetings between the PACU and ward staff and the OR manager. In addition, two new beds were made available in the High Dependency Unit.

The causes underlying staff busyness were found to be multifactorial in nature. Instances included the unavailability of porters, the necessity of lunch breaks, and staff engagement in handover processes during transitions of shift duties. Furthermore, delays were observed in certain cases due to issues pertaining to patient admissions procedures.

A multifaceted strategy was implemented to mitigate these challenges and attain the predetermined objectives established by the department. This approach encompassed the establishment of a collaborative team comprised of personnel from various key departments, notably including members from the Post-Anesthesia Unit, the Operation Theater Manager, Consultant Anesthetists, chief anesthesia residents, and senior ward staff, including shift supervisors. Regular monthly meetings improved communication among the team members, thereby contributing significantly to the department’s attainment of its established performance benchmarks.

### Limitations:

This audit exhibits certain limitations. Firstly, it focuses primarily on non-clinical factors affecting PACU discharge, potentially neglecting crucial clinical variables. Secondly, it lacks exploration of patient perspectives and experiences, which could offer valuable insights. Ultimately, it remains imperative to longitudinally observe the department’s capacity to sustain adherence to established benchmarks over the next few years.

## CONCLUSION

Patient care in the PACU involves monitoring and managing complications until discharge readiness. Clinical and non-clinical factors can contribute to delays, necessitating a multifaceted approach and improved communication for efficient discharge processes.

## References

[1] Ryan J, Doster B, Daily S, Lewis C (2017). Key performance indicators across the perioperative process:holistic opportunities for improvement via business process management. Bus Process Manag J.

[2] Samad K, Khan M, Hameedullah, Khan FA, Hamid M, Khan FH (2006). Unplanned prolonged postanaesthesia care unit length of stay and factors affecting it. J Pak Med Assoc.

[3] Schulz EB, Phillips F, Waterbright S (2020). Case-mix adjusted postanaesthesia care unit length of stay and business intelligence dashboards for feedback to anaesthetists. Br J Anaesth.

[4] Ego BY, Admass BA, Tawye HY, Ahmed SA (2022). Magnitude and associated non-clinical factors of delayed discharge of patients from post-anesthesia care unit in a comprehensive specialized referral hospital in Ethiopia, 2022. Ann Med Surg (Lond).

[5] Cobbe KA, Barford-Cubitt S (2018). Nonclinical factors affecting PACU discharge:a clinical audit in a one-day surgery unit. J Perianesth Nurs.

[6] Morris AM, Hoke N (2015). Communication is key in the continuum of care. OR Nurse.

[7] Fixler T, Wright JG (2013). Identification and use of operating room efficiency indicators:the problem of definition. Can J Surg.

[8] Yee MS, Tarshis J (2023). Anesthesia quality indicators to measure and improve your practice:a modified Delphi study. BMC Anesthesiol.

